# Developmental Differences in the Structure of Executive Function in Middle Childhood and Adolescence

**DOI:** 10.1371/journal.pone.0077770

**Published:** 2013-10-29

**Authors:** Fen Xu, Yan Han, Mark A. Sabbagh, Tengfei Wang, Xuezhu Ren, Chunhua Li

**Affiliations:** 1 Department of Psychology, Zhejiang Sci-Tech University, Hangzhou, China; 2 State Key Laboratory of Cognitive Neuroscience and Learning, Beijing Normal University, Beijing, China; 3 Psychology Department, Queen’s University, Kingston, Canada; 4 Goethe University Frankfurt, Frankfurt a.M., Germany; 5 Beijing No. 12 High School, Beijing, China; University of Missouri-Kansas City, United States of America

## Abstract

Although it has been argued that the structure of executive function (EF) may change developmentally, there is little empirical research to examine this view in middle childhood and adolescence. The main objective of this study was to examine developmental changes in the component structure of EF in a large sample (N = 457) of 7–15 year olds. Participants completed batteries of tasks that measured three components of EF: updating working memory (UWM), inhibition, and shifting. Confirmatory factor analysis (CFA) was used to test five alternative models in 7–9 year olds, 10–12 year olds, and 13–15 year olds. The results of CFA showed that a single-factor EF model best explained EF performance in 7–9-year-old and 10–12-year-old groups, namely unitary EF, though this single factor explained different amounts of variance at these two ages. In contrast, a three-factor model that included UWM, inhibition, and shifting best accounted for the data from 13–15 year olds, namely diverse EF. In sum, during middle childhood, putative measures of UWM, inhibition, and shifting may rely on similar underlying cognitive processes. Importantly, our findings suggest that developmental dissociations in these three EF components do not emerge until children transition into adolescence. These findings provided empirical evidence for the development of EF structure which progressed from unity to diversity during middle childhood and adolescence.

## Introduction

Executive function (EF) is the ability to monitor and regulate different types of cognition and behavior to achieve specific internal goals [1]–[2]. EF serves as an umbrella term that includes multiple processing components, such as attentional control, cognitive flexibility, set-shifting, inhibition, intentional control, purposive action, set maintenance, working memory, and planning [3]–[5]. Among these components, updating working memory (UWM), inhibition, and shifting are the most widely researched EF processes [2], [6]–[8], because they are lower-level (i.e., supposedly implicated in complex executive components, such as planning), and relatively well-defined [2]. UWM (often termed working memory by most authors) refers to the processes involved in monitoring and updating representations in working memory by adding new relevant information and deleting no-longer-relevant information [9], [2]. Inhibition is the ability to deliberately suppress the prepotent (i.e., habitual, dominant, autonomic) responses when those actions run counter to goal achievement [10], [2]. Shifting is the ability to flexibly switch between mental sets, mental operations or different task rules [11], [2].

Although theoretically dissociable, these three aspects of EF may share some cognitive substrates. Over the last decade, a number of studies have investigated the EF structure with respect to these three components with the goal of determining whether they are indeed separable, or they are best thought of as a mostly unitary cognitive process. For adult, these investigations have concluded that though moderately correlated, UWM, inhibition, and shifting can vary independently which suggests that they may indeed be separable. This pattern has been called the full three-factor structure [2], [12]–[13]. However, in childhood, especially during middle childhood and adolescence, not all studies replicated this finding [14]–[22]. Furthermore, few studies, if any, investigated whether the factor structure of EF changes across age groups. Thus, the main goal of this study was to investigate the developmental differences of the structure of these three EF components during middle childhood and adolescence.

The three-factor structure of UWM, inhibition, and shifting was first proposed by Miyake et al. based upon data from adults [2]. In order to examine the distinctiveness of these three EF components in college students, Miyake et al. used relatively simple tasks that were thought to tap each of the three main factors of EF separately, such as running memory, Stroop, number-letter, to index UWM, inhibition, and shifting respectively. Performance on these tasks was then submitted to confirmatory factor analysis (CFA), to extract latent variables capturing the unique covariances among the tasks in each factor battery. Using CFA, they compared models with one, two, or three factors. The results indicated that the full three-factor model was the best fit model relative to models with fewer factors. They concluded that UWM, inhibition, and shifting were indeed distinguishable, yet correlated EF components, namely diversity of EF. Since then, evidences from both behavior studies [12]–[13] and neuroimaging studies [23]–[24] have supported the claim that UWM, inhibition, and shifting are diverse.

Intriguingly, the three-factor structure obtained in adults has not been replicated in young children [14]–[17]. A series of studies conducted by Wiebe and colleagues found that in a sample of children between 2 and 6 years of age, the tasks tapping inhibition and working memory loaded on a single latent factor, that is, inhibition and working memory were not separable [14]–[16]. Similar to the findings of Wiebe and colleagues, Hughes, Ensor, Wilson, and Graham found that a single-factor structure best captured the relationship among working memory, inhibition, and planning at the ages of 4 and 6 in a longitudinal study [17].

Contrary to the studies with young children, previous research focused on middle childhood and adolescence has reported mixed results [18]–[22]. In a group of 7–9-year-old children, Brydges, Reid, Fox, and Anderson observed that a single-factor model was sufficient to account for performances on a battery of tasks tapping UWM, inhibition, and shifting [18]. However, some researchers found that with slightly older children, two of the three executive components might be distinguishable [19]–[20]. For example, in children aged 9 to12 years, a two-factor model including UWM and shifting best accounted for EF performance after controlling for what they called non-executive factors (i.e., naming factor, participants were required to rapidly name geometrical figures, digits, or letters in tasks loading the naming factor) [19]. A two-factor model was also suggested for 11–12 year olds by St Clair-Thompson and Gathercole who used exploratory factor analysis to identify two executive factors: inhibition and UWM [20]. Finally, some research that included still older children has provided evidence for a three-factor model [21]–[22]. For instance, the study by Wu et al. supported the three-factor structure including UWM, inhibition, and shifting in 7–14-year-old children [21]. Similar results were reported by Lehto, Juujärvi, Kooistra, and Pulkkinen in a sample of children aged 8 to 13 years [22].

From this brief review of the developmental work, it appears that one potential reason for the mixed results is because the studies used participants from different age spans [18]–[20]. The studies that find for one-factor structure tend to include much younger children than the studies that find evidence for three-factor structure. To date, studies have not been able to address the possibility that there may be developmental changes in the factor structure of EF. Most of the research with school-aged children collapsed participants across the age range they studied, for example, from middle childhood to post-adolescence [19], [21]–[22]. Doing so may have obscured important developmental changes in the factor structure of EF that we might predict to be present based upon findings. Moreover, UWM, inhibition, and shifting have different developmental trajectories during that period, specifically improve quickly during middle childhood, and gradually mature through adolescence [25]–[28]. A second possible reason for the mixed results is that different studies used different measures to assess the same latent factor. For example, the latent factor inhibition was indicated by Eriksen Flankers task and Go/no-go task in some studies [18], but by Tower of London and Matching Familiar Figures Test in other studies [22].

Given the developmental differences [25]–[28], it is necessary to examine the factor structure of EF across age groups. To date, there have been just two studies along these lines, with disparate findings. In one, Huizinga, Dolan, and van der Molen selected four age groups (7 years old, 11 years old, 15 years old, and 21 years old) to investigate the relationships of UWM, inhibition, and shifting. They did not find that the factor structure of EF changed with age in their study, only UWM and shifting were separable in all age groups, even in the adult group [28]. In the second, Shing, Lindenberger, Diamond, and Davidson divided children and adolescents into three age groups (4–7 years, 7–9.5 years, and 9.5–14.5 years), and found that the factor structure of EF gradually separated with age. More specifically, memory maintenance and inhibitory control were not separable in 4–7 year olds and 7–9.5 year olds, but they were separable in 9.5–14.5 year olds [29]. Their results are difficult to integrate with others, because they used memory maintenance rather than UWM.

Thus, in the current study, we examined the developmental differences of the structure of UWM, inhibition, and shifting in middle childhood and adolescence. Recently, some researchers argued that the degree of unity or diversity of EF structures may be different at different age groups [26], [30]. According to this view and previous findings [18]–[22], [29]–[30], we hypothesized the developmental trend in the factor structure of EF may be from a one- or two-factor model to a three-factor model across development. Specifically, on the basis of previous findings found in 7–9 year olds [18],[29] and adult sample [2],[12], we hypothesized a one-factor model of EF (that incorporates UWM, inhibition, and shifting) might best explain the performance of 7–9 year olds, but that the full three-factor model may best explain the performance of older children aged 13–15 years old.

Following the approach of Miyake and his colleagues [2],[12], we used tasks that were designed to tap a single EF component while placing minimal demands on other EF components. Performance on these tasks was then submitted to CFA to characterize the fit of five possible models, which include the traditional three-factor model proposed by Miyake and colleagues, one one-factor model, and three two-factor models (see Table 1). To characterize developmental changes in the factor structure of EF, EF tasks were administrated to children and adolescents aged 7–15 years old who were divided into three age groups (7–9 years old, 10–12 years old, and 13–15 years old). This age range and group division was chosen because it captures the period over which previous studies have suggested that the factor structure of EF changes from having a one-factor structure (7–9 years old) [18],[29] to a more diverse structure [25],[29]. Finally, because CFA is a large sample technique (sample size should be at least 100), and the use of larger samples tend to provide more precise and stable factor structure, the sample size in each age group was relatively large in comparison to other studies (n = 140–165).

**Table 1:** Five alternative models tested in this study.

Models	
1. Full three-factor	Three EF components are separable, though correlated
2. One-factor	Three EF components are not separable. All tasks tapping UWM, inhibition, and shifting load on a single latent factor
Two-factor models	
3. UWM & Inhibition-Shifting collapsed	UWM is separable from inhibition and shifting; inhibition and shifting are not distinguished
4. Shifting & Inhibition-UWM collapsed	Shifting is separable from inhibition and UWM; inhibition and UWM are not distinguished
5. Inhibition & Shifting-UWM collapsed	Inhibition is separable from shifting and UWM; shifting and UWM are not distinguished

Note. UWM, updating working memory.

## Methods

### Ethics Statement

Guardians and teachers were given a letter explaining the purpose of this study. Written informed consent was obtained from each participant’s guardian. All procedures were approved by the ethics committee of the State Key Laboratory of Cognitive Neuroscience and Learning, Beijing Normal University.

### Participants

A total of 457 children and adolescents were recruited from three primary schools and three junior middle schools of a large-sized town located in east China. All participants received a notebook as gift for their participation. According to the previous findings [18],[25],[29], the sample was divided into three age groups: 7–9 years (M = 8.78 years, SD = 0.57, 73 boys, n = 140), 10–12 years (M = 11.59 years, SD = 0.88, 84 boys, n = 165), and 13–15 years (M = 14.41 years, SD = 0.86, 76 boys, n = 152). Most participants came from rural areas of China, where most families belonged to low to middle socioeconomic status (SES) group. The highest education level of their parents was as follows: approximately 4.3% of parents had earned an undergraduate education degree, 3.3% had received a junior college education degree, 18.5% had gotten a high/technical secondary school diploma, 59.8% had received a junior high school diploma, and 14.3% had a diploma of primary school. We used the non-verbal Raven Standard Progressive Matrices (China Revised edition, SPM-CR, 1989) [31] to assess participants’ intelligence. Based on the norm of the Chinese version for each age group, raw scores of the SPM-CR were converted to standardized scores. All participants had normal intelligence on the basis of the standardized scores of the SPM-CR, with mean standardized scores between 70.23 and 84.98 (SD, between15.36 and 26.51) in 7–9 year olds, between 60.58 and 72.09 (SD, between 15.36 and 26.51) in 10–12 year olds, and between 60.25 and 73.80 (SD, between 18.41 and 29.77) in 13–15 year olds.

### EF Measures

All participants completed a battery of EF tasks to tap the three EF components. The tasks were selected based on two principles. First, tasks chosen in the present study should be sensitive to the development differences of UWM, inhibition, and shifting during middle childhood and adolescence. Second, we used relatively simple tasks that were designed to especially tap one of the three executive components while placing minimal demands on others to avoid the problem of task impurity. Specifically, n-back tasks and running memory tasks were employed to measure UWM. Both tasks involve constantly monitoring and updating information in working memory. The tasks used to tap inhibition were the go/go-go task and color-word Stroop task. Both tasks require deliberately inhibiting prepotent responses. The tasks used to tap shifting were number-pinyin task and dots-triangles task. Both tasks require shifting between mental sets. Because of possible cultural differences between western in which these tasks were originally developed, and the current context (rural China), we adapted those classic tasks as necessary to ensure that they were suitable for Chinese children. All EF tasks were computerized, and were programmed in E-Prime (Version 1.2, Psychological Software Tools).

#### Measures of UWM

N-back task. There were two conditions in the n-back task, 1-back and 2-back, using the same stimulus materials (adapted from Jaeggi, Buschkuehl, Jonides and Perrig, 2008) [32]. In each condition, a small purple square was presented at one of eight different locations within a bigger white square. In the 1-back condition, the participants were told to press the “same” key if the location where the small purple square appeared was the same as the stimulus shown immediately prior to this square and to press the “different” key if the location at which the small purple square was presented was different from the one prior. In the 2-back condition, participants were instructed to press the “same” button whenever the stimulus at hand matched the one presented 2 positions back in the sequence and to press “different” whenever the locations were mismatched. In each condition, there were 16 practice trials followed by 42 target trials. Each stimulus was presented for 3000 ms, followed by an 800-ms interstimulus interval (ISI). The main dependent variable was the proportion of correct responses.

Running memory task. This task was adapted from Van der Sluis et al. [19]. In this task, participants were presented with series of digits successively, which varied in length from 3, 5, 7, to 9 digits. Participants were asked to constantly recall the last 3 digits presented to them. To ensure continuous updating, participants were asked to say aloud the last 3 digits they had seen, regardless of where they were in the sequence. Participants achieved this by adding the latest digit to a cluster and dropping the oldest one, and then saying the new cluster of 3 digits out loud. For example, if the digits presented were “5, 7, 3, 1, 6”, the participants should have read “5…57… 573…731…316”, and then recalled “316” at the end of the trial. The length of the sequences varied unpredictably for the participants. The task consisted of 12 target sequences (3 lists of each sequence length), resulting in 84 clusters of digits to be recalled. Before the target list began, there were 3 practice sequences (1 of length 3, 5, 9). Digits within a sequence were presented serially with 1000-ms per digit, followed by 1000-ms blank screen. The score was the proportion of digit clusters recalled correctly.

#### Measures of inhibition

Go/no-go task. This task was adapted from Eigsti et al. [33]. Participants were instructed to press the spacebar as quickly as possible whenever a target (go) stimulus (a square with a left diagonal) was presented (75% of trials) but to inhibit the response when the non-target (no-go) stimulus (a square with a vertical line in the middle) appeared on the screen (25% of trials). To measure the development of inhibition in the sample whose age was older than 6 years, the task included three conditions: the number of go trials before a no-go trial varied as either 2, 4, or 6. The more go trials that precede a no-go trial, the more difficult it is to inhibit the preponderant response generated by go trials. The duration of each stimulus was 500 ms with an ISI ranging from 800 to 1200 ms. This task consisted of 96 experimental trials and 15 practice trials. The proportion of successfully inhibited no-go stimuli was the dependent measure.

Color-word Stroop task (Chinese character version) [34]. In this task, the four keys (Z, X, N, M) on the keyboard represent the four colors (red, yellow, blue, and green). Participants were required to name the color of a stimulus by pressing the key corresponding to the color on each trial. There were three conditions: (1) in the baseline condition, participants must name the ink color of patches colored red, blue, yellow, or green; (2) in the incongruent condition, there is a mismatch in the semantic and the color of Chinese characters (e.g., Chinese character for “red” shown in green ink), and the participants were asked to judge the printed color of a word while ignoring its meaning; (3) in the congruent condition, the color-words were printed in congruent ink colors (e.g., Chinese character for “red” shown in red ink), and participants also need to judge the name of color of a word. This task included 24 baseline trials in block 1, and 24 incongruent trials with 6 congruent trials in block 2. The participants also received two blocks of 20 trials (8 baseline trials in block 1, 10 incongruent trials and 2 congruent trials in block 2) for practice. Stimuli were presented for 3500 ms, and the ISI was 800 to 1200 ms. The dependent variable was the reaction time (RT) difference between the trials in the baseline condition and the trials in the incongruent condition.

#### Measures of shifting

Number-pinyin task. This task was derived from number-letter task used by Rogers and Monsell [35] and Miyake et al [2]. In the original number-letter task, participants switched between classifying numbers (2, 4, 6, and 8 for even; 3, 5, 7, and 9 for odd) and classifying English letters (G, K, M, and R for consonant; A, E, I, and U for vowel). In the current study, we replaced English letters with Chinese pinyins (b, p, m, and f for the initial consonant of a Chinese syllable; α, e, i, and ü for the simple vowel of a Chinese syllable) since Chinese pupils are not familiar with the vowels and consonants in English. A number-pinyin pair (e.g., 3e) was presented in one of the four quadrants on the screen. The participants were instructed to judge whether the number was odd or even when the number-pinyin pair was presented in one of the bottom quadrants whereas they were asked to judge whether the pinyin was a consonant or vowel when the pair was presented in one of the top quadrants. This task consisted of three blocks. The number-pinyin pair was always presented in one of the bottom quadrants for the first block (30 target trials, 12 practice trials), always in the top quadrants for the second block (30 target trials, 12 practice trials), and in the clockwise rotation around all four quadrants for the third block (60 target trials, 20 practice trials) in which the participants had to shift between the number task and the pinyin task. The duration of each number-pinyin pair was 6500 ms. After a response was given, the next number- pinyin pair was presented. The time interval between the response and the next pair was 1000 ms. The dependent variable was the difference between the average RTs on alternation trials in the third block and the average RTs of the trials from the first two blocks.

Dots-triangles task. This task was adopted from Huizinga et al. [28]. Different numbers of red dots or green triangles were presented in a 4×4 grid, and there were three to eight dots or triangles per half of this grid. In the *dots* task, the participants were instructed to decide whether there are more dots in the right or left (in block 1, 30 target trials and 10 practice trials). During the *triangles* task, the participants had to judge whether there are more triangles in the top or bottom (in block 2, 30 target trials and 10 practice trials). In the third block, four “dots” tasks and four “triangles” tasks appeared alternately (72 target trials and 16 practice trials). Each dot or triangle pattern was presented in the middle of the screen for 3500 ms until a response was given and was then followed by 1000 ms ISI. Similar to the Number-Pinyin task, the dependent variable was the difference between the average RTs of the alternative trials in the third block and the average RTs of the trials from the first two blocks.

### Procedures

All tasks were administered in a quiet classroom. All participants were tested for non-verbal Raven standard progressive matrices in groups of 8–30 students for 30–40 minutes. The running memory task was administered individually for all participants. The remaining EF tasks were tested individually for 7 and 8 year olds and simultaneously in groups of two for 9–15 year olds. Participants sat at separate tables and used different computers during simultaneously testing procedures. EF measures took place in two sessions. Each session lasted approximately 30–40 minutes for a total of 60–80 minutes. The tasks administered in Session 1 included the Stroop, dots-triangles, 1-back, and 2-back. Tasks in Session 2 included the go/no-go, number-pinyin, and running memory. The interval time of the two sessions was approximately 1 week. The order of the two sessions was counterbalanced across participants. Psychology graduate students who were trained prior to testing administered the tests.

### Outliers

In each executive task, missing data were identified for any accuracies and RTs that exceeded 3 standard deviations in each age group. We also performed bivariate outlier analyses on the correlations among these tasks designed to tap the three EF components. Specifically, outliers were identified by computing leverage, student t, and Cook’s D values. Three participants were removed due to these analyses (i.e., levers values>0.05, t values> |3.00|, or Cook’s D values >1.00). Moreover, we performed a two-stage trimming procedure for three tasks (Stroop, number-pinyin, and dots-triangles) in which RT acted as the dependent variable. First, all incorrect trials and trials shorter than 150 ms (too fast to be meaningful) were excluded from the analysis for each participant. Second, we followed the same procedures used in previous studies [28],[35]. If the accuracy of performance was less than 55% in one of the conditions in these three tasks, the corresponding dependent variables of those tasks were coded as missing (baseline condition and incongruent condition in Stroop task, alternative condition and repetition condition in dots-triangles task and number-pinyin task). After the trimming procedures above, the missing values amounted to 7.4% for 7–9 year olds, 4.2% for 10–12 year olds, and 3.6% for 13–15 year olds.

### Statistical Analysis

Analysis of covariance (ANCOVA) was used to assess age effects on each measure with gender as a covariate. To assess the structure of UWM, inhibition, and shifting, CFA was performed in Mplus version 6.0 [36] with the maximum likelihood method. According to past studies, we used multiple fit indices to evaluate the fit of each theoretical model, including chi-square (χ^2^), χ^2^/df, root-mean-square error of approximation (RMSEA), comparative fit index (CFI), and Akaike Information Criterion (AIC) [37]. Models were considered to be good fits with non-significant χ^2^ values at the 0.05 level, χ^2^/df of less than 2, CFI of more than 0.90, RMSEA of less than 0.08, and smaller AIC values [38]. In all of the following analyses, the directionality of the dependent variables on the basis of RT was reversed so that higher scores indicated better performance.

## Results

### Descriptive Statistics and Significance Test of Development for each Task

The mean scores, standard deviations, skewness, and kurtosis for performance on each executive task for each age group were listed in table 2. All variables showed normal distributions in each age group (i.e., absolute value of skewness <2 and kurtosis <7) [39]. Cronbach’s alpha or the split-half (odd–even) correlation was computed as index of internal consistency for each of the variables. As is shown in table 2, all estimates were higher than 0.78, indicating reasonable reliability. The zero-order correlations among these executive tasks were generally low (*r* = 0.36 or lower) in each age group (see Appendix).

**Table 2:** Descriptive statistics and age group differences on all executive tasks in each age group.

	7–9 years old			10–12 years old			13–15 years old			ANCOVA			
Tasks	M(SD)	Ske	Kur	M(SD)	Ske	Kur	M(SD)	Ske	Kur	F	P	η^2^	Reliability
1-back (%)	86.21(10.58)	−1.42	1.90	91.12(6.36)	−1.18	2.09	93.41(4.69)	−.51	−.06	33.13	<.001	.13	.82^a^
2-back (%)	58.64(13.68)	.44	−.32	66.30(13.83)	.08	−.70	71.22(13.77)	−.28	−.76	29.52	<.001	.12	.78^a^
Running Memory (%)	69.99(12.36)	.04	−.47	80.90(10.21)	−.25	−.85	86.66(9.81)	−.69	−.20	85.92	<.001	.28	.84^a^
Go/no-go (%)	50.21(15.62)	−.02	−.69	58.42(18.06)	−.12	−.68	69.37(17.85)	−.46	−.52	43.10	<.001	.16	.91^b^
Stroop (ms)	415.47(190.62)	.18	−.41	309.48(185.82)	.58	−.01	230.29(115.01)	.55	.36	41.57	<.001	.16	.94^b^
Number-pinyin (ms)	782.88(307.07)	.04	.90	722.03 (284.28)	.46	.19	608.42(236.76)	.88	.20	15.28	<.001	.01	.83^b^
Dots-triangles (ms)	611.46 (328.82)	.48	−.07	591.55(284.37)	.65	.19	543.05(289.69)	.74	.20	20.59	<.001	.08	.92^b^

Note. Ske,Skewness, Kur, kurtosis.

a Reliability was calculated using Cronbach’s alpha.

b Reliability was calculated by adjusting split-half (odd–even) correlations with the Spearman-Brown prophecy formula.

For the 2-back, preliminary analyses suggested that the gender difference reached a significant level, though the effect size was small. Moreover, gender did not interact with age on any tasks. Even so, we used gender as a covariate in follow-up analysis to ensure that the effect of age would not be affected by gender. Analysis of covariance (ANCOVA) with gender as a covariate resulted in significant main effects of age on all executive tasks, *F* = 3.17∼85.92, all *p*<0.05, *η^2^* = 0.01∼0.28 (see table 2). The findings showed that all executive tasks used in the present study were sensitive to the developmental differences in EF across the three age groups. In addition, post-hoc Bonferroni tests showed that the performance for the 1-back, 2-back, running memory, go/no-go, and Stroop tasks was better in 13–15 year olds than in 10–12 year olds, and better in 10–12 year olds than in 7–9 year olds. However, for the performance on the number-pinyin and dots-triangles tasks 7–9 year olds did not differ from 10–12 year olds, but 13–15 year olds significantly outperformed 10–12 year olds.

### Confirmation Factor Analysis

Multi-group CFA was carried out to evaluate whether the factor structure of EF performance was the same across the three age groups. Guided by existing research, we first established the best fitting model for the entire sample (7–15 year olds). The results of CFA showed that the fit of the full three-factor model was better than the one-factor model and two-factor models (see table 3), suggesting that these three executive components were distinguishable from each other in 7–15 year olds. Next, to assess whether the three-factor construct was applicable to each age group (7–9 year olds, 10–12 year olds, and 13–15 year olds), multi-group CFA models were established in the three age groups. First, a configural invariance model was established as the baseline model for the multi-group comparison. In this baseline model, the configuration of factor loading was set to be identical for each age group, while parameters (e.g., specific factor loadings, factor variance, etc.) were free to vary across age groups. This baseline model, however, was not successful and the minimum requirement of multi-group comparison was not met. This suggested that the three-factor construct may be appropriate for one of the three age groups but not for every age group.

**Table 3:** Goodness of fit indices for alternative CFA models for total sample.

Models	χ^2^	df	P	χ^2^/df	RMSEA	CFI	AIC
**1. Full three-factor** **model**	**19.89**	**11**	**.05**	**1.81**	**.04**	**.97**	**71.74**
2. One-factor	62.04	14	.00	4.43	.09	.86	104.04
Two-factor							
3. UWM & Inhibition-Shifting collapsed	28.08	13	<.01	2.16	.05	.96	100.98
4. Shifting & Inhibition-UWM collapsed	37.16	13	<.01	2.86	.06	.93	100.31
5. Inhibition & Shifting -UWM collapsed	29.02	13	<.01	2.23	.09	.78	98.52

Note. The best fitting model is indicated in bold. UWM, updating working memory,

RMSEA, root-mean-square error of approximation; CFI, comparative fit index; AIC, Akaike Information Criterion.

**Figure 1:**
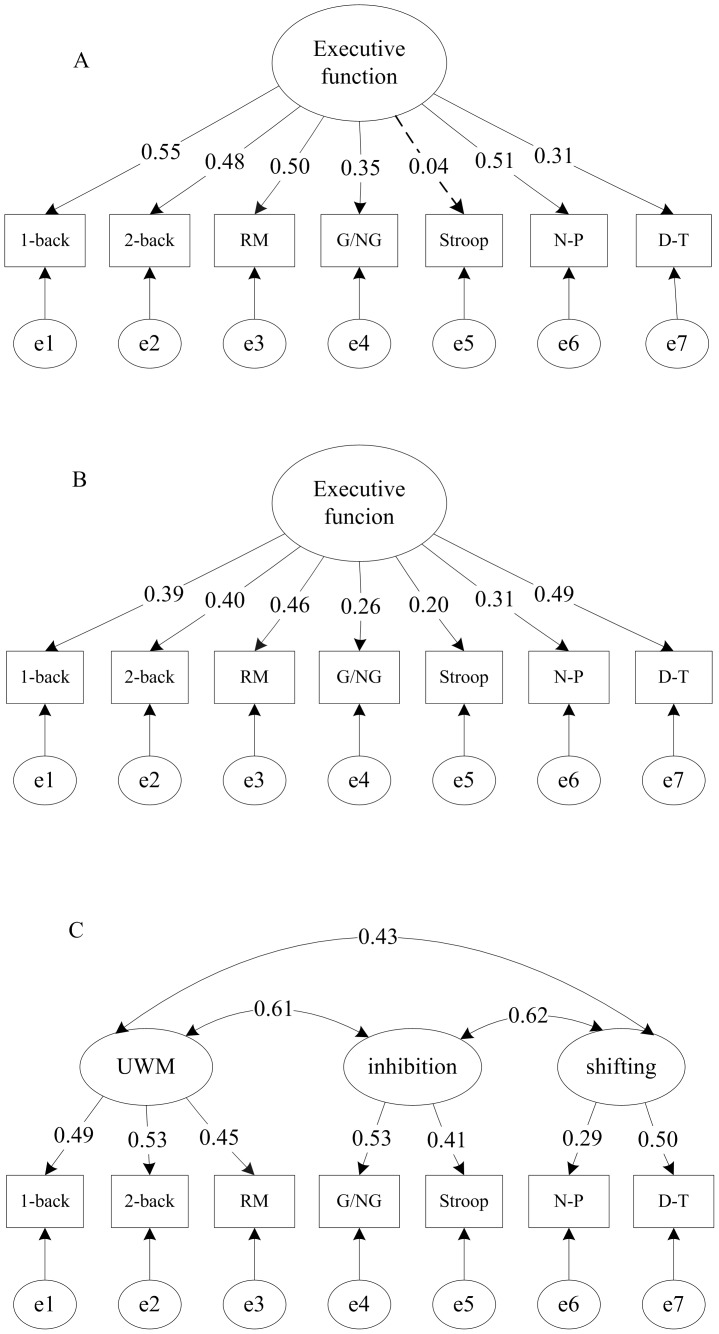
Best fit model in each age group. RM = running Memory, G/NG = go/no-go, N–P = number-Pinyin, D–T = dots – triangles. All standardized parameters were significant (P<0.05) except the loading of the Stroop task (dotted line) in 7–9 year olds. A for 7–9 years old, B for 10–12 years old, C for 13–15 years old.

According to our hypothesis that the factor structure of EF may be different at different age groups, the five alternative models were tested for each age group separately using CFA. Table 4 presents the fit results of these models in each age group. In 7–9 year olds, both the one-factor model and full three-factor model were acceptable models according to the fit indexes (see table 4). In that case, we should select the simpler model based on parsimony principle [14],[37], so the one-factor model (Model 2) was preferred. Furthermore, according to AIC which can be used to compare competing models [40], the one-factor model is preferred because it has smaller AIC than the full three-factor model (see table 4). The results suggested that UWM, inhibition, and shifting were not statistically dissociable in 7–9 year olds, that is, the structure of EF was unitary in this age range (see figure 1 A). The same was true for 10–12 year olds where the CFA results again suggested that a one-factor model (model 2) provided the most parsimonious account of EF performance (see table 4), which suggested that the unitary construct was still appropriate for describing the relationship of the three components in 10–12 year olds (see figure 1B). The single latent factor was named Executive Function. For the 13–15 year olds, however, there was a shift for them, the three-factor model (model 1) provided the best fit of the five models (see table 4). These findings suggest that the latent variables of UWM, inhibition, and shifting each constituted statistically separable component of 13–15 year old children’s EF performance. In other words, for the older group, the three EF components were clearly distinguishable, though they are moderately correlated with each other for children aged 13 and older (see figure 1 C).

**Table 4:** Goodness of fit indices for alternative CFA models in each age group.

	Models	χ^2^	df	P	χ^2^/df	RMSEA	CFI	AIC
7–9 years old	*1. Full three-factor	14.38	12	.37	1.20	.03	.96	59.34
	**2. One-factor**	**15.65**	**14**	**.34**	**1.12**	**.03**	**.95**	**56.64**
	Two-factor							
	3. UWM & Inhibition-Shifting collapsed	23.87	13	.03	1.84	.08	.68	67.87
	4. Shifting & Inhibition- UWM collapsed	N.A.						
	5. Inhibition & Shifting -UWM collapsed	16.27	13	.24	1.25	.04	.89	60.27
10–12 years old	1. Full three-factor	22.10	11	.02	2.01	.08	.81	68.15
	**2. One-factor**	**19.24**	**14**	**.30**	**1.37**	**.05**	**.95**	**57.44**
	Two-factor							
	3. UWM & Inhibition-Shifting collapsed	26.80	13	.01	2.06	.08	.71	70.80
	4. Shifting & Inhibition- UWM collapsed	19.71	13	.11	1.52	.06	.90	61.86
	5. Inhibition & Shifting -UWM collapsed	28.10	13	.01	2.16	.08	.68	67.74
13–15 years old	**1. Full three-factor**	**15.72**	**11**	**.15**	**1.43**	**.05**	**.95**	**63.72**
	2. One-factor	32.51	14	.00	2.32	.09	.74	74.51
	Two-factor							
	3. UWM & Inhibition-Shifting collapsed	24.05	13	.03	1.85	.08	.85	68.05
	4. Shifting & Inhibition- UWM collapsed	24.95	13	.03	1.88	.08	.83	68.49
	5. Inhibition & Shifting -UWM collapsed	29.02	13	.01	2.23	.09	.78	73.02

Note. The best fitting model is indicated in bold. UWM, updating working memory, RMSEA, root-mean-square error of approximation; CFI, comparative fit index; AIC, Akaike Information Criterion. N. A., not admissible.

* not positive definite residual covariance matrix.

We further examined whether the regression coefficients (factor loadings) of the observed indicators of Executive Function (the single latent factor) were identical in both 7 to 9 years of age and 10 to12 years of age. To address this issue, multi-group CFA was conducted. We first established a configural invariance model. The fit of this model displayed acceptable fit to the data, χ^2^ = 29.90, χ^2^/df = 1.07, CFI = 0.98, RMSEA = 0.02. Then, we nested within the configural invariance model, a metrical measurement invariance model in which the factor loadings were constrained to be equal across the two groups in addition to the loading patterns. The fit of the metrical measurement invariance model was not satisfactory, particularly with respect to the value of CFI, χ^2^ = 42.96, χ^2^/df = 1.21, CFI = 0.87, RMSEA = 0.04. A direct chi-square comparison also indicated that there was a significant difference between the configural invariance model and the metrical measurement invariance model (Δχ^2^ = 13.06, Δ*df* = 6, *p<*0.05), suggesting that the factor loadings differed between the two younger age groups (see figure 1A and figure 1B). Executive Function (the single latent factor) explained 30%, 23%, 25%, 12%, and 26% of the variance in the 1-back, 2-back, running memory, go/no-go, and number-pinyin, respectively, in 7–9 year olds, but only 15%, 16%, 21%, 7%, and 10% of the variance in 10–12 year olds.

## Discussion

The present study investigated the developmental changes in the factor structure of EF. We administrated a battery of appropriate measures tapping UWM, inhibition, and shifting in a large sample of Chinese children and adolescents, and then used CFA to characterize the latent factor structure of EF among three age groups: 7–9 years, 10–12 years, and 13–15 years. The results of CFA indicated that over development, the factor structure of EF changed from a one-factor to a full three-factor model. Specifically, a single factor accounted for EF performance in both 7–9 years and 10–12 years, though even between these periods there was some development in the specific factor loading patterns. For the oldest group, however, a three-factor model best accounted for the performance of UWM, inhibition, and shifting. That is, the three EF components separated into three distinct components at 13–15 years of age. Put another way, the results of CFA indicated that the structure of the three components developed from unity to diversity. Our findings provide evidence for the age differentiation hypothesis which postulates that the structure of cognitive abilities develops from a relatively unified, general ability to more differentiated, specific cognitive abilities with child development [41]–[42].

Our results are similar to those of Shing et al. [29], who also found that the specific executive components gradually separated across age groups. However, Shing et al. only examined the separation of memory maintenance and inhibitory control, and found that they were separable in 9.5–14.5 years. In the current study, we found that the constructs of UWM, inhibition, and shifting were distinct at 13–15 years of age but not earlier. The slight inconsistency between these results may come from the different tasks chosen in the two studies. Specifically, the tasks used in the study by Shing et al. only assessed set maintenance whereas ours included the requirement to actively update working memory representations. It is well known that active updating of working memory has a protracted developmental time course relative to maintenance [43], which may delay the differentiation of EF structure in our study.

However, the specific timing of our findings dovetails nicely with documented changes in cortical functioning that are happening around the same time in development. EF is often linked to the functioning of the prefrontal cortex (PFC) [8]–[9], and the development of EF appears to be closely linked to the development of PFC [29]. The PFC goes through dynamic structural and functional changes around the age of 12. From the structural perspective, longitudinal neuroimaging studies have demonstrated that gray matter in the PFC increased throughout childhood with a maximum size occurring at approximately 12 years, followed by a sharp loss after 12 years of age [44]. At the functional level, there are critical changes in the patterns of PFC activation that are elicited during EF performance, including enhanced activation in critical regions and attenuation in others between 9 years and 12 years [45]. These changes usually result in more focal and less diffuse PFC activation as children transition from childhood to adolescence. The structure and function of PFC change around 12 years old may be associated with the separability of EF structure found in present study.

The results of this study run counter to those of Huizinga et al. [28] who found evidence of only the two latent factors (UWM and shifting) which were distinguishable in children as well as adults. The discrepancy between those findings and our own could be attributed to the characteristics of the three inhibitory tasks. The three inhibition tasks used in their study (stop-signal, Eriksen flanker, and smiley pictures) measured three different types of inhibitory abilities, so a common inhibition factor could not be extracted, which also led to the inconsistent results in the adult sample between their study and past adult studies [2]. However, the two tasks (go/no-go, color-word Stroop) used in our study were specifically designed to rely on proponent response inhibition and little else [46], so a common inhibition factor can be extracted in 13–15 years. Also, the age divisions used by Huizinga et al. differed from ours, and, perhaps most important, the sample size in each age group in the study of Huizinga et al. was limited, especially the youngest age group (n = 71).

There were some limitations of our study that should be addressed in future research. One limitation concerns the missing data, especially in the young age group. After following standard practices for data trimming, 7.4% of 7–9 year olds data was missing. This result may be because the three EF components undergo rapid improvements during childhood and adolescence, so individual differences were considerable. Second, the participants in the present study came from lower SES families. Recent evidence has shown that children from low SES families experience slower EF development relative to children in higher SES categories, especially in working memory and inhibitory control [47]–[48]. The slow development of cognitive abilities may delay the extent to which those abilities are statistically separable from a single EF factor [41]. In any case, studies with children from diverse SES backgrounds may help to establish the generalizability of the patterns we reported here. Finally, for the youngest group, performance on the Stroop task did not load on the single latent factor (Executive Function) in 7–9 year olds, which was not expected. However, the Stroop task loaded on the single latent factor in one-factor model for the 10–12 year olds, and inhibition factor in the three-factor model for the 13–15 year olds. Given these limitations, it would be worthwhile to confirm the findings using different tasks, and extend this work with different EF components in future study.

To conclude, using a relatively large sample, the present study found that the factor structure of EF changed with children’s transition from middle childhood to adolescence. In particular, a single-factor model better accounted for younger children’s EF performance, whereas a three-factor model considering UWM, inhibition, and shifting as separate latent variables provided the best fit for older children’s performance. Our findings further confirmed the view that the underlying nature of children’s EF skills vary with age, and suggest that the neurocognitive systems that support different aspects of EF become increasingly specific and dissociated with age.

## Supporting Information

Table S1 **Correlations between executive tasks for each group.** Note. * *p*<.05, **p<.01.
(DOC)
