# Supplementary material for: Developmental Differences in the Structure of Executive Function in Middle Childhood and Adolescence
Source: PLoS One. 2013 Oct 29;8(10):e77770. doi: 10.1371/journal.pone.0077770 (PMC3812181; doi:10.1371/journal.pone.0077770)
Supplement: Table S1 — Correlations between executive tasks for each group. Note. * p<.05, **p<.01. (DOC) [file pone.0077770.s001.doc]

|  | EF tasks | 1 | 2 | 3 | 4 | 5 | 6 |
| --- | --- | --- | --- | --- | --- | --- | --- |
| 7-9 years old | 1. 1back | - |  |  |  |  |  |
|  | 2. 2back | .36** | - |  |  |  |  |
|  | 3. Running Memory | .19* | .27** | - |  |  |  |
|  | 4. Go/no-go | .16 | .25** | .28** | - |  |  |
|  | 5. Stroop | .12 | .06 | .15 | .13 | - |  |
|  | 6. Pinyins-digits | .10 | .20* | .26** | .24** | .14 | - |
|  | 7. Dots-triangles | .21* | .28** | .32** | .21* | .12 | .18 |
| 10-12 years old | 1. 1back | - |  |  |  |  |  |
|  | 2. 2back | .30** | - |  |  |  |  |
|  | 3. Running Memory | .15 | .36** | - |  |  |  |
|  | 4. Go/no-go | .17* | .21* | .14 | - |  |  |
|  | 5. Stroop | .19* | .24** | .20* | .14 | - |  |
|  | 6. Pinyins-digits | .25** | .29** | .23** | .19* | .19* | - |
|  | 7. Dots-triangles | .14 | .11 | .15 | .23** | .04 | .11 |
| 13-15 years old | 1. 1back | - |  |  |  |  |  |
|  | 2. 2back | .32** | - |  |  |  |  |
|  | 3. Running Memory | .24** | .30** | - |  |  |  |
|  | 4. Go/no-go | .34** | .27** | .16 | - |  |  |
|  | 5. Stroop | .20* | .16* | .20* | .25** | - |  |
|  | 6. Pinyins-digits | .02 | .18* | .15 | .28** | .33** | - |
|  | 7. Dots-triangles | .18* | .22* | .17* | .25** | .10 | .21* |
